# Selective blue-filtering spectacle lens protected primary porcine RPE cells against light emitting diode-induced cell damage

**DOI:** 10.1371/journal.pone.0268796

**Published:** 2022-05-24

**Authors:** Wing Yan Yu, Samantha Sze Wan Shan, Yamunadevi Lakshmanan, Francisca Siu Yin Wong, Kai Yip Choi, Henry Ho Lung Chan

**Affiliations:** 1 Laboratory of Experimental Optometry (Neuroscience), School of Optometry, The Hong Kong Polytechnic University, Hong Kong SAR, China; 2 The Centre for Myopia Research, School of Optometry, The Hong Kong Polytechnic University, Kowloon, Hong Kong SAR, China; 3 Centre for Eye and Vision Research, Hong Kong SAR, China; Saarland University, GERMANY

## Abstract

This study aimed to investigate whether use of a selective-blue-filtering (S-BF) lens can protect cultured primary porcine RPE cells against photo-irradiation. Transmittance of S-BF and UV-filtering (UVF) lenses was characterised spectrophotometrically. RPE cells were exposed to 1700 lux of white (peak λ at 443 and 533 nm; 0.44 mW/cm^2^) or blue (peak λ at 448 and 523 nm; 0.85 mW/cm^2^) LED light for 16 h to evaluate the influence of light source on the culture. The effect of the S-BF and UVF ophthalmic lenses on RPE cell cultures under blue light irradiation was then investigated. Cell viability was compared using trypan blue and MTT assays. Intracellular ROS production was detected by a fluorescein probe CM-H2DCFDA. Expression levels of catalase and Prdx3 were analysed by western blot. Trypan blue staining showed blue light caused more cell death than no light (p = 0.001) or white light (p = 0.005). MTT assay supported the hypothesis that exposure to blue light damaged RPE cells more severely than no light (p = 0.002) or white light (p = 0.014). Under blue light, use of the S-BF lens, which blocked 17% more blue light than the UVF lens, resulted in higher cellular viability (S-BF: 93.4±1.4% vs UVF: 90.6±1.4%; p = 0.022; MTT: 1.2-fold; p = 0.029). Blue and white light both significantly increased ROS production. The S-BF lens protected cells, resulting in lower levels of ROS and higher expression of catalase and Prdx3. To conclude, blue LED light exposure resulted in significant cytotoxicity to RPE cells. Partial blockage of blue light by an S-BF lens led to protective effects against retinal phototoxicity, which were mediated by reduction of ROS and increased levels of antioxidant enzymes.

## Introduction

The wavelength of blue light, also known as high energy visible light, ranges from 400 to 500 nm. It is present both outdoors from the sun and indoors from many artificial light sources, including light-emitting diode (LED) lamps, compact fluorescent tubes, and other digital devices, for instance computer screens, tablets, and smartphones. Compared to incandescent lamps, these new artificial light sources, especially LED, emit a much greater proportion of blue light, which raises concern over the exposure limit. Typically, compact fluorescent tubes emit 25% blue light, cool white LED at least 35%, and incandescent lamps approximately 3% [1].

Periodic light exposure, specifically to blue light and ultraviolet (UV) radiation, induces phototoxic and oxidative damage to the retina, which may contribute to pre-mature ocular aging and development of retinal degeneration, specifically age-related macular degeneration (AMD) [2–4].The crystalline lens normally protects the retina against the phototoxic effects of UV and blue light [5], but during aging, the lens becomes progressively more opaque and yellowish, causing an increase in light absorption in the range of 300 to 500 nm [6]. Indeed, pseudophakic eyes with clear intraocular lenses that only block UV light, have an increased risk for the development and progression of AMD [7, 8].

Numerous studies have investigated the protective effect of blue and UV filters on the retina. It is known that acute exposure of the retina to strong light increases cellular senescence and death both *in vitro* and *in vivo* [2, 9, 10]. Recent research revealed that LEDs at domestic lighting levels also induced retinal injury [11]. Use of blue filters has revealed promising effects in protecting the retina from oxidative stress *in vitro* and in animal models [12, 13]. However, human data using blue-blocking intraocular lenses remains inconclusive [14, 15]. This may be attributable to the complex pathogenesis of retinal degeneration and various lifetime light exposures of individuals. Despite a lack of solid clinical evidence, theoretical and experimental evidence indicate that blue light exposure causes damage to the retina and possibly plays a role in the pathogenesis of AMD [16]. It is therefore advocated that early or asymptomatic AMD patients consider using yellow intraocular lenses for cataract surgery to minimise progression [16].

Eyecare professionals often recommend the use of tinted or filtering lenses as a form of protective eye wear to prevent bright sunlight or high energy visible light from damaging or causing discomfort to the eyes. The most commonly recommended lenses are UV filters, which protect the eyes from UV radiation that can cause photokeratitis, snow blindness, cataracts, pterygium, and retinal degeneration. Due to the tremendous increase in the amount of electronic device screen time over recent years, people have become more aware of the need to protect the eyes against high energy visible blue light. In terms of protection, yellow spectacle lenses theoretically work the best as they filter almost all blue light from entering the eye. However, blue filters (yellow lenses) have potential disadvantages in negatively affecting color discrimination, scotopic vision, circadian rhythms, memory, and cognitive performance [14].

In recent years, the use of selective blue-filtering (S-BF) lenses, which selectively filter the blue-violet high energy light and UV light, were advocated for protection of the eyes. These lenses maintain excellent transparency and allow adequate beneficial blue light to pass through, so that visual functions, such as color discrimination and night vision, are not as disturbed as compared to use of blue filters [17]. To date, supportive evidence for the use of these lenses to protect or delay ocular deterioration is limited. This study aimed to compare the viability of primary cultured porcine retinal pigmented epithelial (RPE) cells under different light conditions and evaluate the protective effect of two lens types, S-BF and UVF lenses, on primary cultured RPE cells against photo-irradiation. It was hypothesized that short wavelength (blue) light would increase oxidative stress in RPE cells and, by partially blocking blue light, using an S-BF lens would provide a protective effect against retinal phototoxicity, that may potentially be useful in preventing the development and progression of AMD.

## Materials and methods

### Isolation and culture of primary porcine RPE cells

Primary RPE cells were isolated from fresh porcine eyes as previously described with modifications [18]. In brief, the extraocular tissues and anterior segment were removed, leaving an eye cup for the dissection of the RPE. The vitreous and sensory retina were then carefully removed from the RPE using forceps. The RPE layer was isolated, washed in phosphate-buffered saline (PBS) and digested in 0.25% collagenase (Sigma-Aldrich, St. Louis, MO, USA) on a shaker at 37°C for 20 min. The digested RPE tissues were pooled, washed in PBS, before being passed through a 40-μm cell strainer (Corning, Shanghai, China). The cells were then washed twice with PBS, centrifuged at 1500 rpm for 8 min and re-suspended in Dulbecco’s Modified Eagle Medium (DMEM) supplemented with 15% fetal bovine serum (FBS), 1% penicillin–streptomycin, and 1% antibiotic-antimycotic (Thermal Fisher Scientific, Rockford, IL, USA). They were cultured for 7 days at 37°C in a humidified atmosphere of 5% CO_2_. The medium was changed three times during the culture period.

After the primary isolation, the cells were subcultured for 7 days at passage 2 and 3 for phenotype characterisation. Some cells at passage 2 were maintained in culture for 5, 7 or 14 days for a separate characterisation. To subculture, the cells were detached from the culture dish by incubation with trypsin-EDTA at 37°C for 5 min. Trypsin action was neutralised by the addition of DMEM with 15% FBS. After centrifugation at 1500 rpm for 5 min, the cells were re-suspended in DMEM supplemented with 15% FBS, 1% penicillin-streptomycin and 1% antibiotic-antimycotic, and seeded on culture plates. They were incubated at 37°C in a humidified atmosphere of 5% CO_2_. Fresh medium was added every other day. Morphology images were captured using an inverted microscope (Eclipse Ti-S, Nikon, Tokyo, Japan).

### Western blot

Porcine RPE cells were lysed with lysis buffer containing 7M Urea, 2M Thiourea, 30 mM TRIS, 1% ASB14, 2% CHAPS (BioRad Laboratories, Hercules, CA, USA) and protease inhibitor cocktail (Roche, Mannheim, Germany). Samples were incubated at 4°C for 1 h, followed by centrifugation at 13,000 rpm for 20 min. The supernatant was obtained, and the protein concentrations measured by the BioRad Protein Assay (BioRad). Twenty-five micrograms of RPE proteins were mixed with loading buffer (0.3 M Tris, 10% SDS, 50% vol/vol glycerol, 3.6 M beta-mercaptoethanol, and 0.5% bromophenol blue), heated at 95°C for 5 min, and then separated on a 10% SDS-PAGE gel before transfer to a polyvinylidene difluoride membrane (BioRad). Non-specific binding was blocked with 5% non-fat dried milk (in 0.1 M Tris-HCl, pH 8.0, 0.5 M NaCl containing 0.05% Tween-20 (TBST)) for one hour at room temperature with gentle agitation. The blot was then incubated with the anti-RPE65 (1:1000; Thermo Scientific), anti-catalase (1:2000; Novus Biologicals, Littleton, CO, USA) or anti-Prdx3 primary antibody (1:5000; abcam, Cambridge, UK) overnight at 4°C. After washing with TBST, the blot was incubated with anti-mouse or anti-rabbit IgG conjugated with horseradish peroxidase (Zymed Laboratories, San Francisco, CA, USA) at room temperature for one hour and visualised by Pierce SuperSignal West Pico Chemiluminescent substrate (Thermo Scientific). The image was captured and analysed with an Azure Imaging System c600 (Azure Biosystems, CA, USA). An anti-actin antibody (1: 10000; abcam) or anti-GAPDH antibody (1:10000; Calbiochem) was used as a loading control.

### Characterisation of LED lights and ophthalmic lenses

The spectral distribution and radiance of white and blue LED light sources were measured by a spectroradiometer (SR-3, Topcon, Tokyo, Japan). The spectral transmittance, within the wavelength range of 200 to 500 nm (step size = 5 μm), of the selective blue-filtering (S-BF) lens and UV-filtering (UVF) lens (referenced with blue filter and Columbia Resin 39 (CR 39) lens) were characterised using a spectrophotometer (Lambda 650S UV/Vis, Perkin Elmer, Waltham, MA, USA). The lens geometric centre was aligned with the measuring axis. These lenses were purchased from ophthalmic lenses suppliers (Corning, USA and Essilor, Paris, France) and of zero prescription power with a refractive index of 1.6.

### LED light irradiation and application of Selective Blue-Filtering (S-BF) and UV-Filtering (UVF) lenses on RPE cell cultures

**Fig 1:**
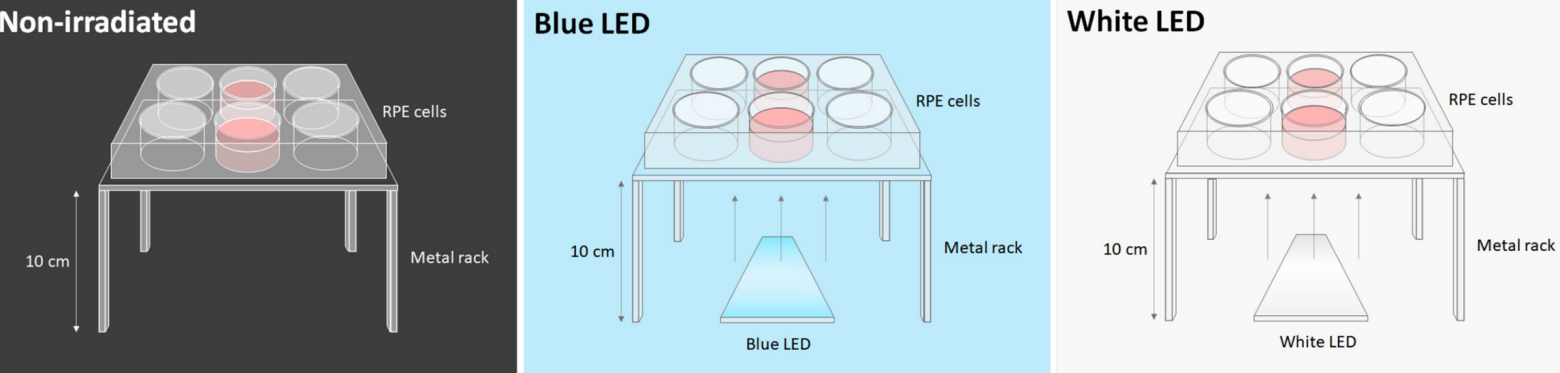
A schematic diagram of the experimental setup in the culture incubator. RPE cells were exposed to 1700 lux of blue (peak λ at 448 nm and 523 nm; 0.85 mW/cm^2^) or white (peak λ at 443 nm and 533 nm; 0.44 mW/cm^2^) LED light for 16 h inside the incubator. Cells were cultured in darkness as a negative control. The culture plates were placed on a metal rack, while the selective blue-filtering (S-BF) and UV-filtering (UVF) lenses were placed beneath the culture plates respectively.

RPE cells cultured on a 6-well plate were exposed to 1700 lux of white (peak wavelengths at 443 nm and 533 nm; 0.44 mW/cm^2^) or blue (peak wavelengths at 448 nm and 523 nm; 0.85 mW/cm^2^) LED light (Hing Lee Electronics Company, Hong Kong) for 16 h inside the incubator. To minimize the effect of light intensity variation across different wells of the 6-well plate, only the central 2 wells were loaded with cell samples. The light source was centred 10 cm underneath the 2 wells. Light was shone from below the well plate, which was kept in the same position for every experiment. A schematic diagram is shown in **Fig 1**. Control cells incubated in the dark and light-treated cells were obtained from the same passage and same eyes as one biological sample to eliminate any pre-existing bias.

The effect of two types of ophthalmic lens on RPE cell cultures under blue light irradiation was then investigated. The cells were exposed as described above, with the addition of S-BF lens or UVF lens (control) beneath the culture plates. Cells were maintained in the dark acted as a control.

### Cellular viability analysis by trypan blue and MTT assay

The viability of the RPE cultured cells was evaluated using trypan blue exclusion test and MTT assay (Sigma-Aldrich). For the trypan blue test, 5 x 10^3^ cells, in DMEM containing 20% FBS, were seeded into each well of a 24-well plate and allowed to adhere for 24 h. After light irradiation for 16 h, the cells were trypsinised, centrifuged at 1500 rpm for 5 min, re-suspended in 0.4% trypan blue solution in serum-free medium for 1 min, and counted by a haemocytometer to determine the percentage of viability.

For the MTT assay, cells prepared in 96-well plates as described above, but at a density of 1 x 10^3^ cells per well, were treated with 5 mg/ml MTT for 4 h at 37°C. Cell viability, defined as the relative amount of MTT absorbance, was measured using a spectrophotometric microplate reader (Model 680, BioRad) at 570 nm.

### Detection of intracellular Reactive Oxygen Species (ROS)

RPE cells were prepared in 96-well plates as described above at a density 1 x 10^3^ cells per well. Ten μM of cell-permeant chloro-methyl 2’,7’-dichlorodihydrofluorescein diacetate (CM-H2DCFDA) (Thermo Fisher Scientific), used as a free radical fluorescein probe, was added to the culture medium and the mixture incubated for 1 h at 37°C. Non-fluorescent in the probe taken up into the cells was converted to its fluorescent form, dichlorodihydrofluorescein (DCFH) following cleavage of the acetate groups and oxidation by intracellular ROS. Fluorescence intensity was then measured at 483 nm against 530 nm as a reference using a Clariostar microplate reader (BMG Labtech, Offenburg, Germany). Cells treated with hydrogen peroxide (H_2_O_2_) at 100 μM were used as a positive control.

### Statistical analysis

All data were obtained from at least three independent experiments performed using three different batches of RPE cells. Data were expressed as mean ± SEM. To evaluate the significant differences, Student’s t-test and One-way ANOVA with Tukey post hoc test were applied accordingly using GraphPad Prism statistical software (San Diego, CA, USA). *p* < 0.05 was considered statistically significant.

## Results

### Characterisation of LED light sources and different spectacle lenses

White and blue LED light had peak wavelengths at 443 nm and 533 nm and at 448 nm and 523 nm, respectively. Although the wavelengths peaks of white and blue LED light were similar, the magnitude of the peaks of these LED light sources differed, with peaks at 448 nm and 523 nm in blue light were about 2-folds and 1.6-folds higher than the respective peaks in white light. Furthermore, the full spectrum of the light the light sources were also different, as that of blue LED light ranged from 450 nm to 600 nm; while that of white light was from 430 nm to 680 nm.

**Fig 2:**
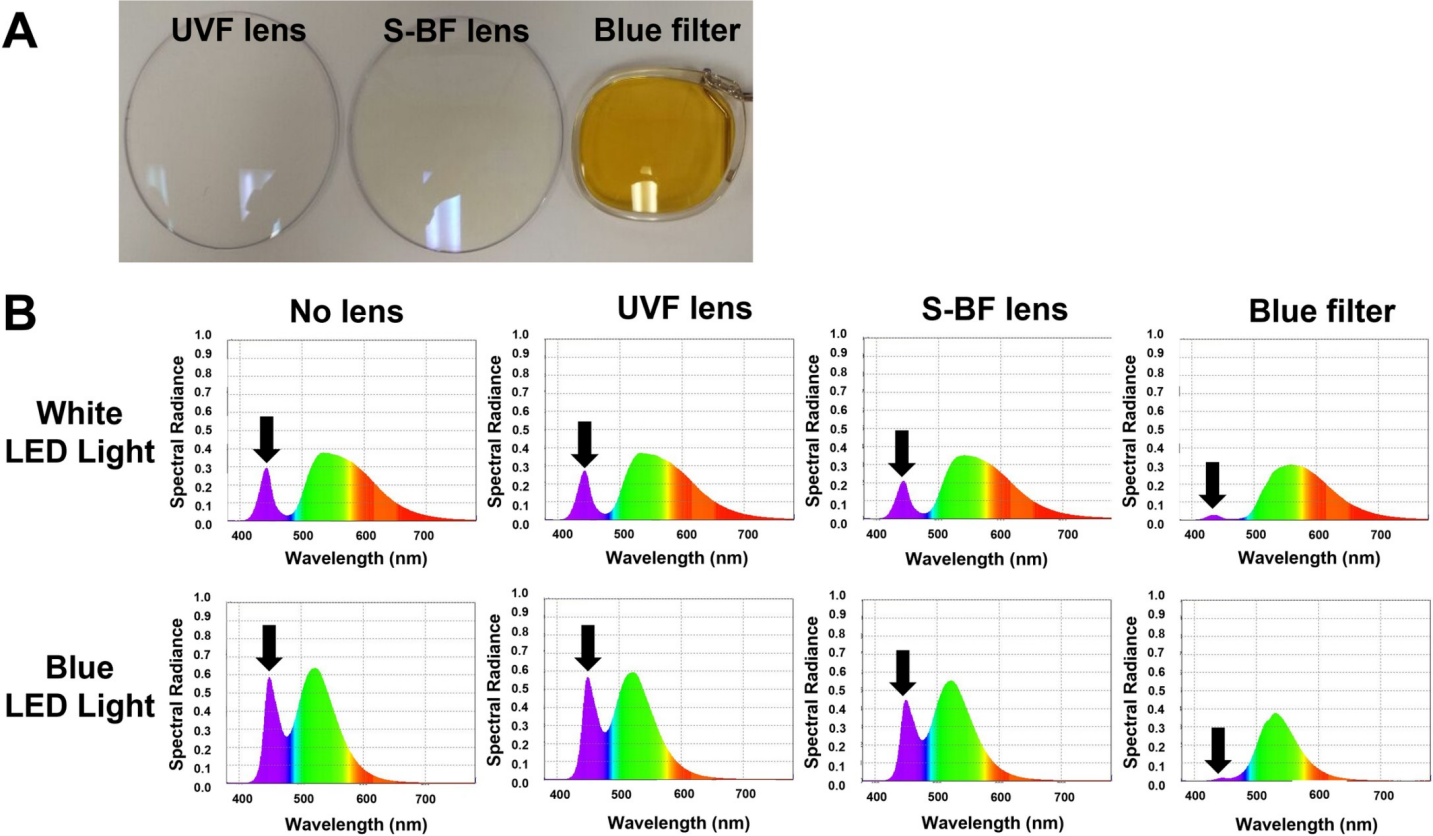
**(A) The appearance of different spectacle lenses.** Selective blue-filtering (S-BF) lens has a light-yellow tint, while UV-filtering (UVF) lens is transparent, and blue blocking filter lens is deeply yellow-tinted. **(B) Spectral radiance of LED lights with and without spectacle lenses.** S-BF lens significantly reduced the blue spectrum (black arrows) under both white or blue LED lights when compared to the UVF lens or without lens condition. Blue filter almost completely blocked the blue spectrum.

**Fig 3:**
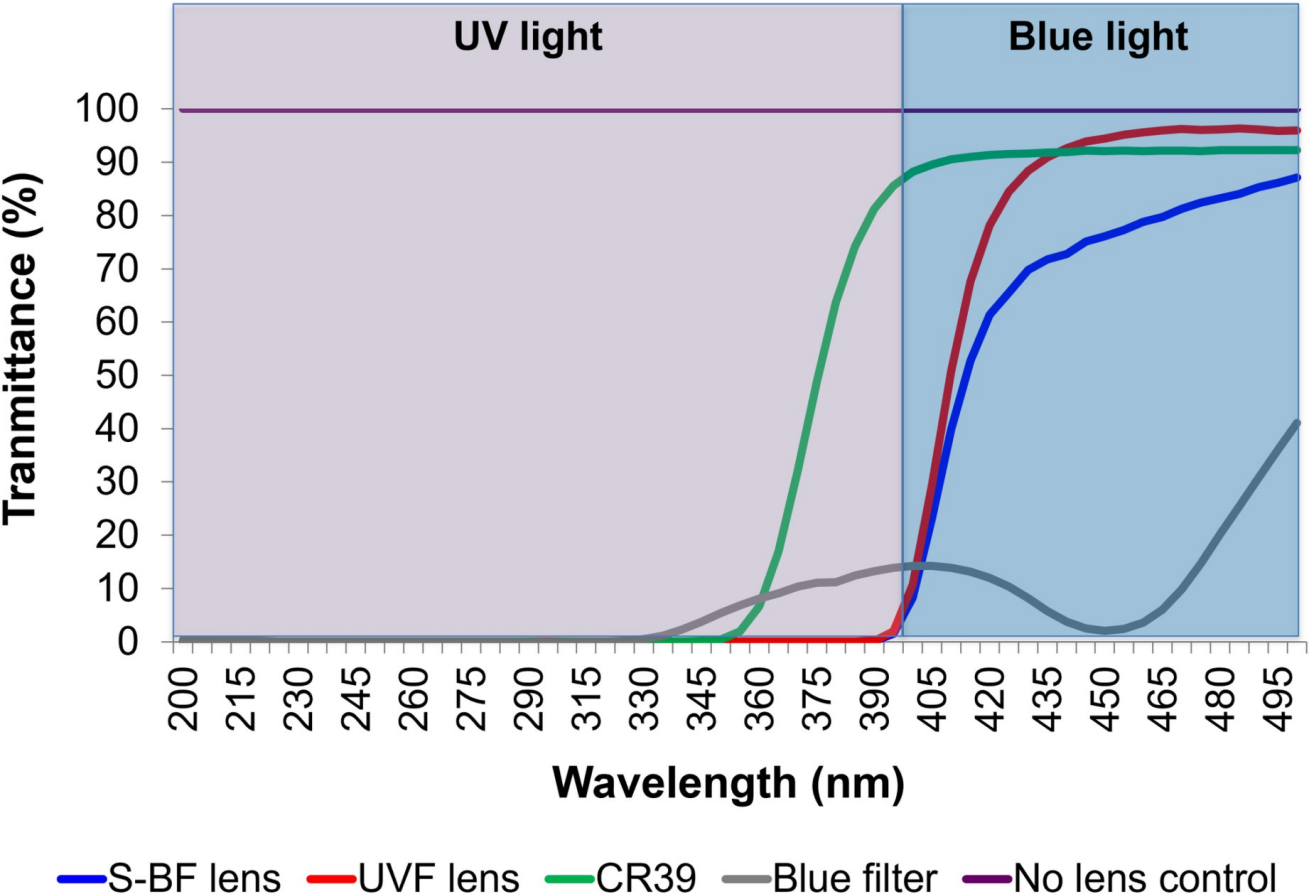
Transmittance of different spectacle lenses as measured by a spectrophotometer. Selective blue-filtering (S-BF) lens reduced 17% more visible blue light compared to the ultraviolet-filtering (UVF) lens, and reduced 35% more blue light compared to the CR39 lens. The blue filter significantly reduced the blue spectrum. The CR39 lens did not have any coating and allowed most blue and some UV light to pass through. A control without any lens allowed 100% transmittance for all wavelengths.

In the presence of an S-BF lens, the spectral radiance of blue light reduced significantly compared to with a UVF lens. The blue filter almost completely blocked the blue spectrum **(Fig 2)**. The average transmittance of S-BF, UVF, CR39, and blue filter lenses from 200 nm to 500 nm was 70.0%, 84.8%, 91.9% and 15.7%, respectively. The S-BF lens reduced 17% more of the visible blue light compared to the UVF lens (area under curve), and 35% more compared to the CR39 lens. The blue filter also significantly reduced blue light transmittance. CR39 lens, which did not have any coating, allowed most blue and some UV light to pass through **(Fig 3)**.

### Characterisation of cultured primary porcine RPE cells

**Fig 4:**
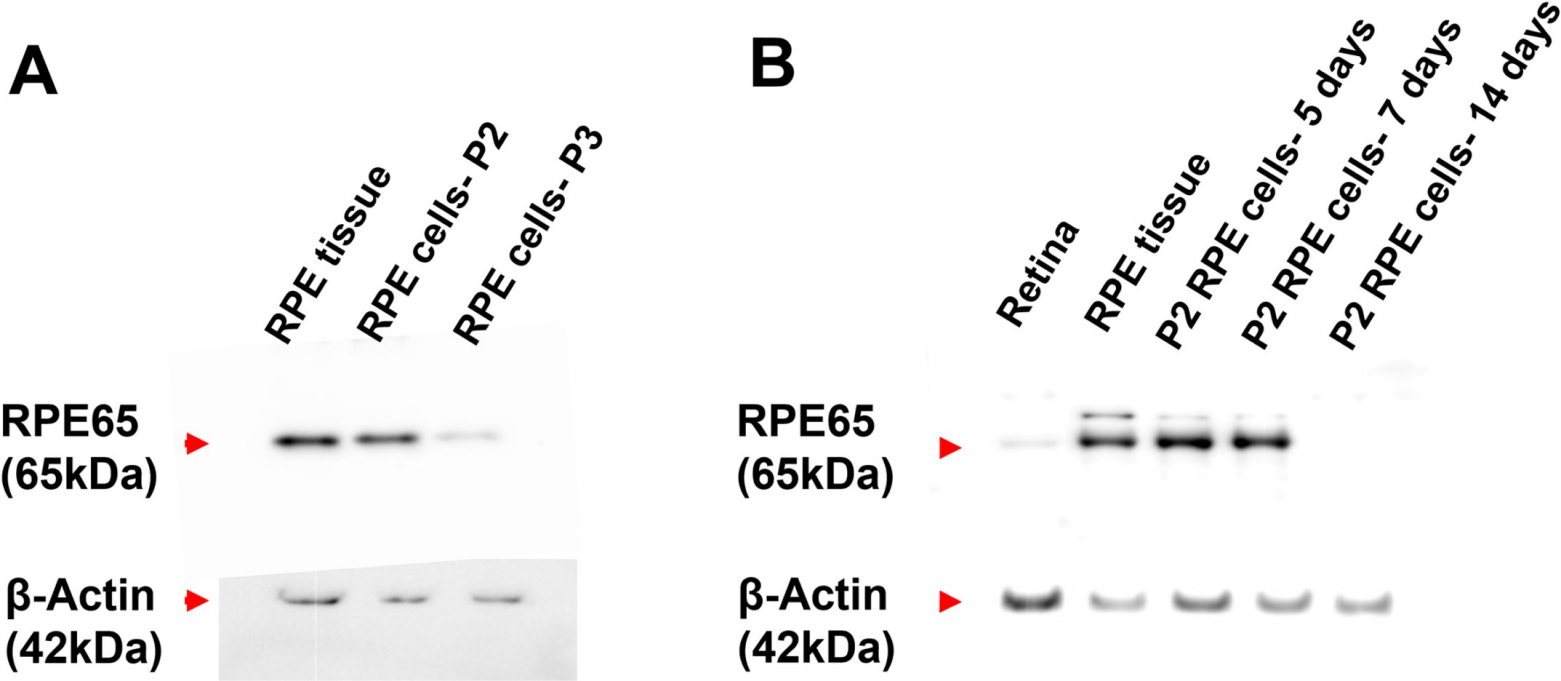
Characterisation of cultured primary porcine RPE cells. **(A)** The expression of RPE marker RPE65 in different passages of the cultured primary RPE cells. After 7 days of culture, passage 2 RPE cells showed higher expression of RPE65 than passage 3. RPE tissue was used as a positive control. **(B)** The expression of RPE65 in passage 2 RPE cells cultured for 5, 7 and 14 days. The passage 2 RPE cells expressed RPE65 up to 7 days, but the expression was absent after 14 days of culture. Whole tissues of RPE and retina were used as positive and negative controls respectively. (n = 3).

The cultured primary porcine RPE cells grew as a monolayer, demonstrating a flat, polygonal morphology. The cultures were free of contamination from scleral fibroblast, which could be easily identified by their slender, elongated appearance, and grew more rapidly as multiple layers upon confluency. Western blot showed that the harvested primary cells at passage 2 cultured up to 7 days expressed RPE65, which is a cell marker for RPE **(Fig 4)**. Passage 2 of the primary RPE cells that were cultured for 7 days were used for all light exposure experiments, as their phenotype characteristics altered after this time point.

### Morphological changes, cell viability and reactive oxygen species production under LED lights

**Fig 5:**
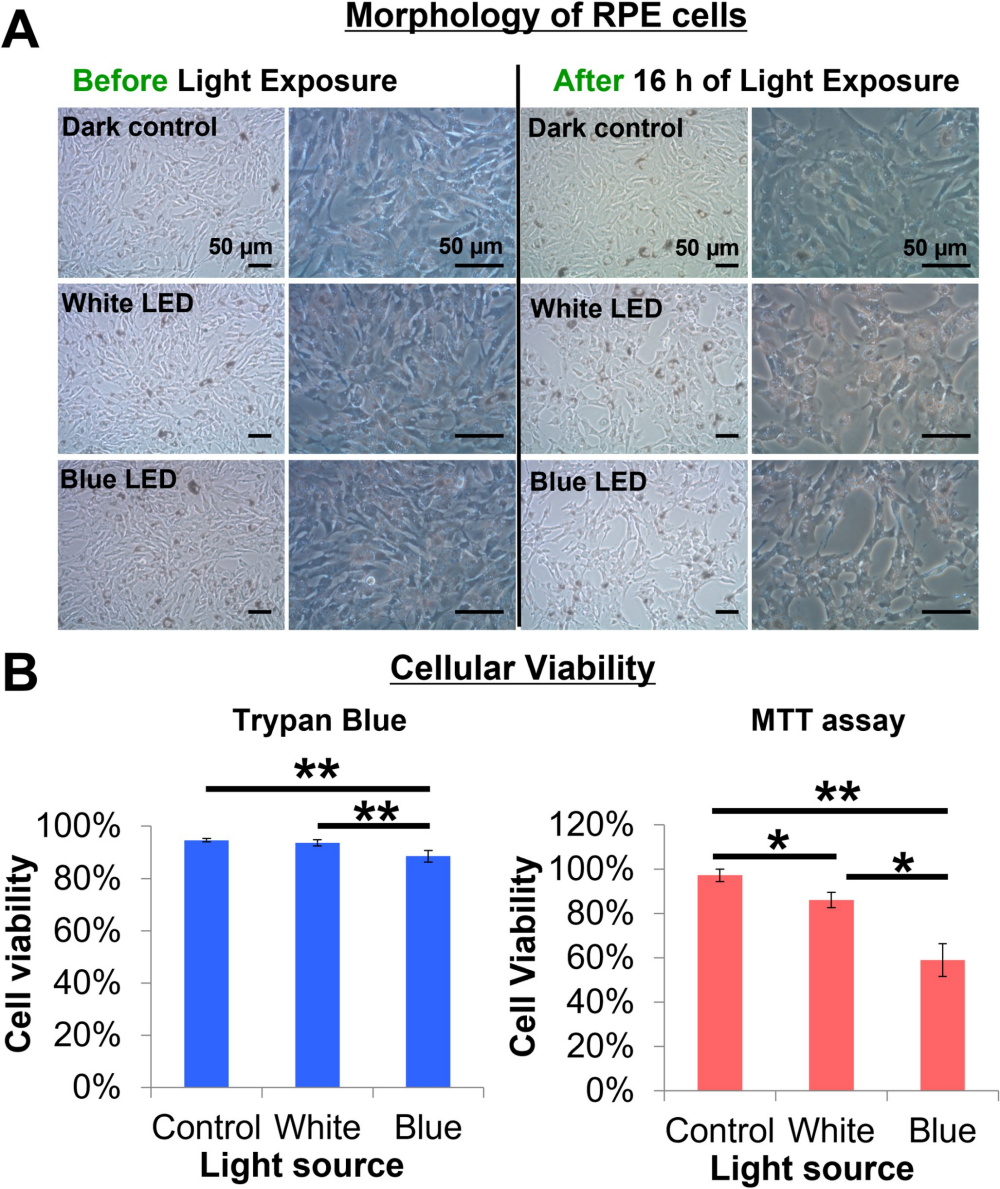
Effect of LED light exposure on the morphology and survival of RPE Cells. **(A)** The RPE cells became more spindle-like in shape, and some detached from the culture plate after 16 h of light exposure. This was more evident in the blue LED irradiated cells. Scale bar represents 50 μm. **(B)** Blue light irradiation significantly induced more cell death compared to white light and the dark control, as shown by trypan blue and MTT assays. Cell viability dropped to 86% and 59% in white and blue LED light, respectively, under MTT assay. (n = 3, one way ANOVA, * p<0.05, ** p<0.01.) The values are expressed as Mean ± SEM.

RPE cells maintained their monolayer structure and polygonal morphology when cultured in the dark. However, they lost their polygonal morphology and became more spindle-shaped after 16 h LED light exposure **(Fig 5A)**. Some even detached from the culture plates. Such cell detachment occurred more frequently under blue LED light.

Trypan blue staining indicated lower cell viability after upon exposure to blue LED light compared to white light (one way ANOVA, p = 0.0051) and dark control (one way ANOVA, p = 0.0010) **(Fig 5B)**. MTT assay also demonstrated that blue light irradiation significantly induced more cell death compared to white light (p = 0.015) and dark control (p = 0.0016). Cell viability dropped to 86% and 59% in blue and white LED light, respectively.

**Fig 6:**
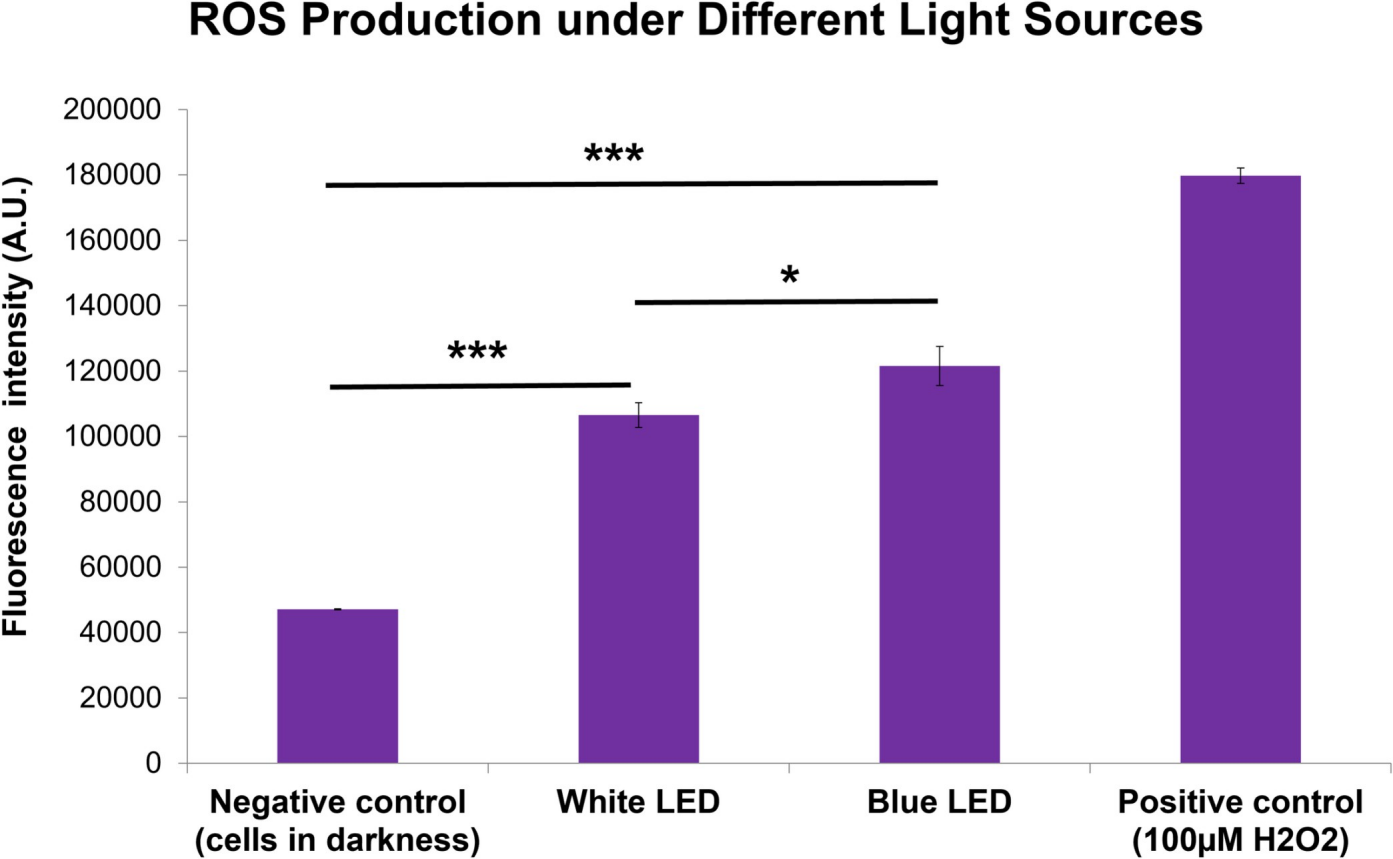
Production of cellular Reactive Oxygen Species (ROS) under different light sources. Blue and white LED lights increased ROS generation compared to controls cultured in darkness (p<0.001). Blue LED light induced a higher ROS production than white LED light (p = 0.021). Hydrogen peroxide at 100 μM was used as a positive control. (n = 3, one way ANOVA, * p<0.05, *** p<0.001.) The values are expressed as Mean ± SEM.

In addition, as illustrated in **Fig 6**, both white (2.3-fold, one way ANOVA, p<0.001) and blue (2.6-folds, one way ANOVA, p<0.001) LED lights significantly induced higher levels of ROS generation compared to the control in the dark. Blue LED light induced higher ROS production than white LED light (one way ANOVA, p = 0.021).

### The effect of an S-BF lens on cellular viability, ROS production, and anti-oxidative enzymes expression

**Fig 7:**
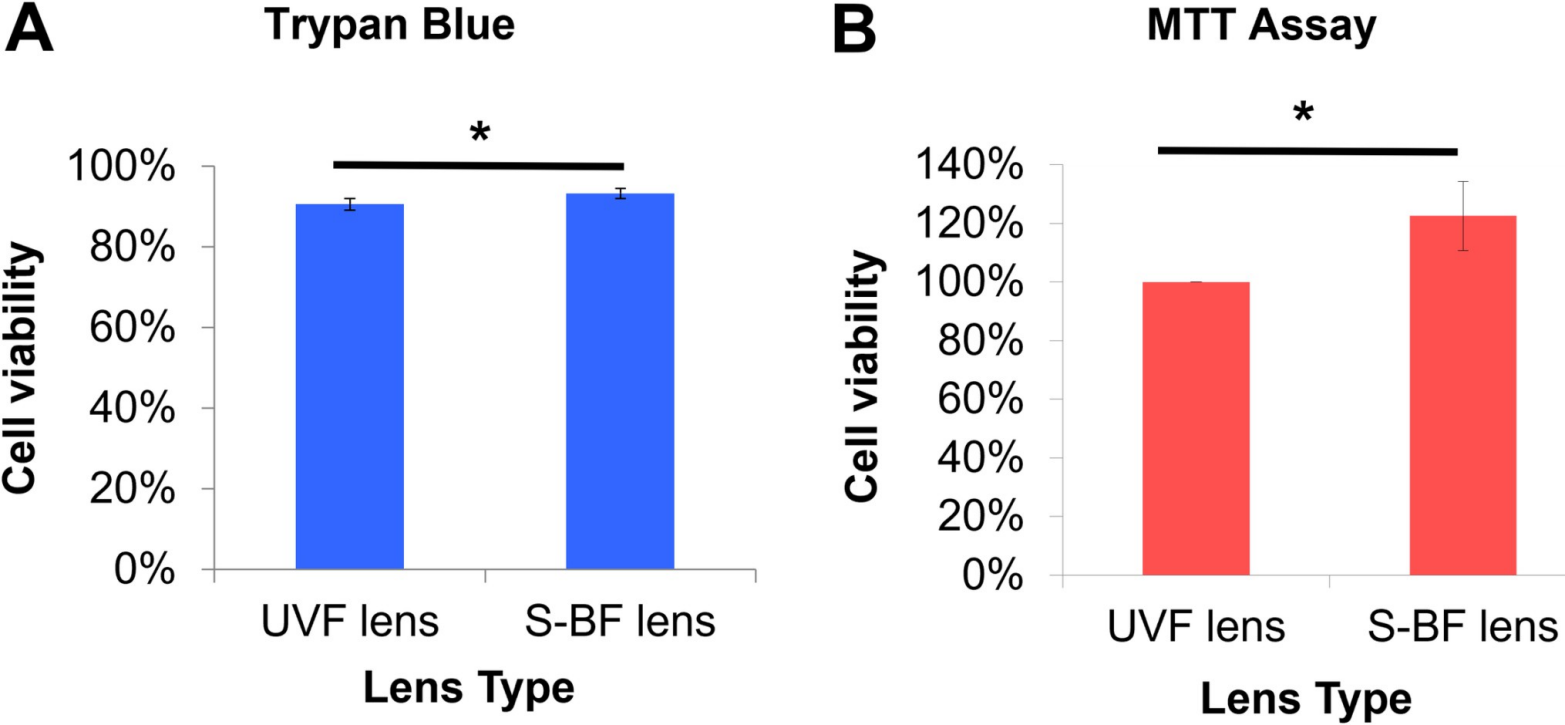
Selective Blue-Filtering (S-BF) lens protected RPE cells from blue light irradiation-induced cytotoxicity. **(A)** Under blue LED irradiation, S-BF lens showed significantly elevated viability compared to the UV-filtering (UVF) lens (93.4±1.4% vs 90.6±1.4%) using trypan blue (p = 0.022) assay. **(B)** MTT assay also revealed that S-BF lens increased cellular viability by 22.5% (p = 0.029). (n = 3, t-test, * p<0.05).

**Fig 8:**
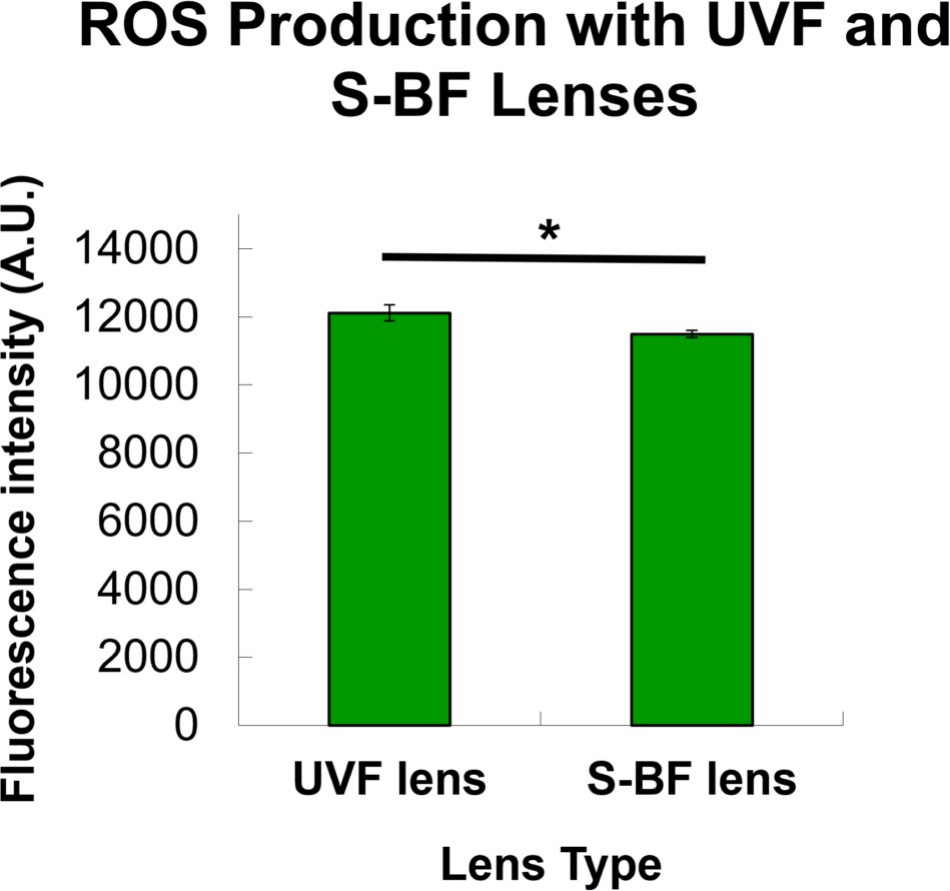
The level of Reactive Oxygen Species (ROS) under Selective Blue-Filtering (S-BF) and UV-Filtering (UVF) lenses. Under blue LED, RPE cells with the selective blue-filtering (S-BF) lens showed a lower level of ROS compared to the UV-filtering (UVF) lens (p = 0.038), indicating less oxidative stress was present when the S-BF lens was in place. (n = 3, t-test, * p<0.05) The values are expressed as Mean ± SEM.

**Fig 9:**
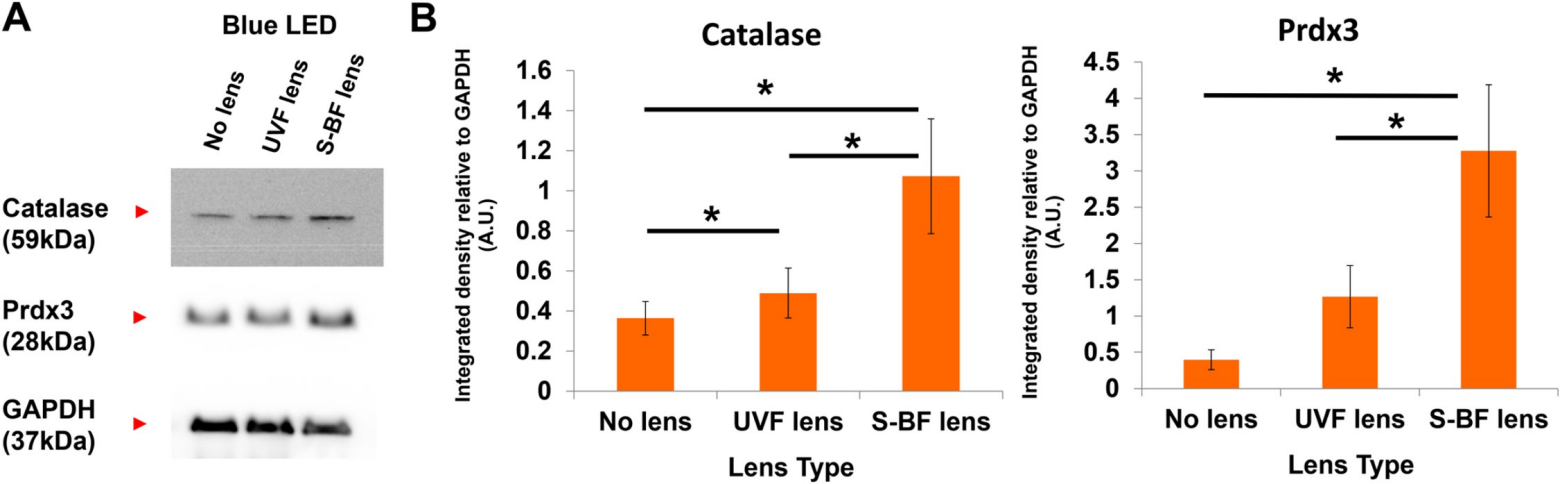
Protein expression of anti-oxidative enzymes in the UV-Filtering (UVF), Selective Blue-Filtering (S-BF) and no lens groups under blue LED irradiation. **(A)** Representative western blot of the anti-oxidative enzymes catalase and Prdx3. GAPDH was used as a loading control. **(B**) Quantification analysis of protein levels of catalase and Prdx3. Under blue light irradiation, S-BF lens showed higher expression of catalase than no lens (p = 0.035) and UVF lens (p = 0.020). UVF lens also showed a higher level of catalase compared to no lens (p = 0.040). The S-BF lens showed a higher expression level of Prdx3 than no lens (p = 0.026) and UVF lens (p = 0.028). A.U.: arbitrary unit. (n = 3, one way ANOVA, * p < 0.05) The values are expressed as Mean ± SEM.

In the presence of an S-BF lens, a higher percentage of RPE cells survived under blue LED irradiation. The trypan blue assay revealed use of an S-BF lens resulted in higher cell viability compared to the UVF lens (93.4±1.4% vs 90.6±1.4%) (t-test, p = 0.022) **(Fig 7A)**. Similarly, MTT assay demonstrated that S-BF lens led to 22.5% higher cell viability (t-test, p = 0.029) **(Fig 7B)**. In terms of ROS production, with the addition of an S-BF lens, the RPE cells under blue LED exposure showed 5.2% less ROS production compared to those with the UVF lens (t-test, p = 0.038), indicating less oxidative stress was present when the S-BF lens was in place **(Fig 8)**. Further investigation of protein expression of anti-oxidative enzymes under blue LED irradiation revealed that S-BF lens showed significantly higher expression of catalase than no lens (one-way ANOVA, p = 0.035) and UVF lens (one-way ANOVA, p = 0.020). UVF lens also showed a higher level of catalase compared to no lens (one-way ANOVA, p = 0.040). The S-BF lens showed a significant higher expression level of Prdx3 than no lens (one-way ANOVA, p = 0.026) and UVF lens (one-way ANOVA, p = 0.028) **(Fig 9)**. The relative protein expression was normalised to GAPDH.

## Discussion

To our knowledge, the present study is the first to demonstrate the protective effect of a commercially available S-BF ophthalmic lens, which partially blocked short wavelength visible light, in particular blue LED light irradiance, on primary cultured porcine RPE cells. The study demonstrated that *in vitro* exposure to blue LED light damaged primary porcine RPE cells more severely compared to white LED light. The LED light-induced cell damage was associated with rapid increase in ROS. Under the irradiation of blue LED light, the S-BF lens substantially reduced ROS formation, increased availability of anti-oxidative enzymes, and preserved cell viability of primary RPE cells as compared with a UVF lens.

To understand their effects on RPE cells and evaluate the effectiveness of S-BF and UVF lenses, LEDs were used as the light source in this study. The domestic white LED lights are usually produced by combining the blue LED with a coating of yellow phosphor, which then delivers the white light sensation [19]. This explains the similarity of the peak wavelength of white (443 nm and 533 nm) and blue (448 nm and 523 nm) LED lights used in the current study, although the spectral radiance is greater in the latter. Upon irradiation, the RPE cells showed an upsurge in ROS levels causing cell loss, which was relatively greater under blue LED light irradiance (41% non-viable cells) than those under white LED light irradiance (14% non-viable cells). In fact, the susceptibility of RPE cells to phototoxicity has been reported previously, using both *in-vitro* cell culture [20–27] and *in-vivo* animal models [27–34]. The cell culture models differed between studies in terms of choice of RPE cells, irradiating light source and its wavelength, and intensity and duration of exposure. While some used human primary RPE cells [25, 35], and a human RPE cell line ARPE19 [22–24, 27, 36], others obtained cells from rabbit [21, 37], rodent [38, 39], or porcine [20] sources, with [21–24, 27, 35–37, 40, 41] or without lipofuscin accumulation [25, 26, 39, 42]. The light sources applied were either tungsten halogen [20, 23, 24, 36, 37, 41, 43], mercury [21–24], mercury-xenon [40, 42], UV [38], fluorescent [44], or LED lamps [25–27] with wavelength ranging from shorter to longer wavelength. It is worth noting that blue [20, 21, 23, 24, 27, 35, 37–39, 41–43] or white [20, 26, 36, 40, 43–45] light irradiance on RPE cells caused cellular damage, when cells were exposed either to an intense light for a brief period [21–24, 36, 40, 43] or a moderate light for a longer duration [20, 25, 27, 35, 38, 39, 42, 44]. More importantly, it was found that the retina and RPE were much more sensitive to blue light damage [10, 20]. Our findings correspond with other cell culture studies in that shorter wavelength radiation induced greater RPE cell damage than longer wavelength light [20, 25, 43], irrespective of the light source.

In the presence of lipofuscin, the RPE cells were even more highly susceptible to phototoxicity [21–24, 27, 35–37, 40, 41, 43, 44]. In addition, such cell damage was also reported in RPE cells that lack A2E chromophore [25, 26, 39, 42]. In the current study, following photo-irradiation, the RPE cells without lens insertion were shown to have significant damage. We have shown an increase in ROS production upon light irradiation, which is supported by other studies [20, 24, 25, 35, 36, 39]. In addition to increased levels of oxidative stress, previous studies have reported that RPE cells under strong light exposure developed mitochondrial and DNA damage [25, 35], altered angiogenic cytokine expressions [26, 27, 36, 40, 44], damaged tight junction structures [27], upregulation of inflammatory cytokines [27], and pro-apoptotic proteins [40], which eventually resulted in apoptotic cell death [22, 23, 39, 42]. The accumulation of such RPE cell damages is considered an early event in the induction of AMD [46].

Subsequently, the present study investigated the protective effects of S-BF and UVF lenses on RPE cells following blue LED light irradiance. While both lenses lowered the transmission of light below 500 nm, the S-BF lens reduced light transmission by nearly 30%, compared to 15% by the UVF lens, and was thus, even though both types of lenses were able to significantly reduce the ROS generation, the S-BF lens was more effective in improving cell viability. The cellular defence mechanisms of the retina to combat ROS typically decline with age [47].The highly oxygenated outer retinal layers, in particular, the RPE and photoreceptors, are considered most vulnerable to photochemical damage. The beneficial effects of S-BF lenses may be useful to prevent or alleviate ocular degeneration by reducing cellular stress responses that may be involved in the development of RPE diseases, such as AMD. Our findings were corroborated by increased levels of anti-oxidative enzymes in S-BF lens-treated RPE cells and help to explain the difference in protective effects observed between the two lens types.

Previous cell culture studies have reported the protective effects against RPE phototoxicity using different types of blue blocking IOLs or filters with varying levels of transmittance. Sparrow and Zhou reported that yellow-tinted IOL (50% transmittance) prevented around 80% of cell damage following blue or white light irradiance of lipofuscin accumulated RPE cells for 20 min [43]. Other non-tinted IOL types with less than 10% reduction in the transmission of short or wide-band light could only inhibit cell death by 10–20% and 20–40% respectively [43]. Rezai and co-workers also showed that yellow-tinted IOL decreased the rate of cell damage by about 50% following chronic blue light irradiance of non-lipofuscin RPE cells for 10 days [42]. Later, Zhou and Sparrow in 2011 applied blue filtering (yellow) ophthalmic lens (absorption spectra of 375 to 450 nm), prepared by mixing the perylene dye at different concentrations (from 1.0 to 35 parts per million) in polycarbonate lens, to study the protective effects against blue light irradiance of lipofuscin loaded RPE cells for 20 min. The results showed a graded preservation of cell viability of 18%, 30% and 47% using MTT assay, when pigment concentrations increased from 3.8, 7.5 and 35 ppm, respectively [41].

Camerro et al. used an LED light source to demonstrate the maximum photodamage induced by short wavelength blue light followed by white and green light when RPE cells were exposed to a 12 h light-dark cycle for 3 days [25]. Their subsequent study also showed that the use of a blue filter reduced the irradiance level of blue LED light from 5.11 mW/cm^2^ to 1.15 mW/cm^2^ and improved cell viability by 30% [45]. Interestingly, few studies have compared the protective effects of a blue filter and UVF IOL against photodamage [36, 40, 44]. It was shown that a blue filter IOL offered better protection than UVF IOL by significantly inhibiting the upregulation of vascular endothelial growth factor [26, 36, 40, 44], increasing the levels of pigment epithelium-derived factor and free glutathione [36], reducing ROS generation [36], upregulating anti-apoptotic protein (Bcl2) and downregulating pro-apoptotic protein (Bax) expression [40]. Overall, the studies showed that full blockage of blue light can significantly inhibit cell death. Although only a selective blockage resulting in by 30% reduction in blue light transmission was applied in this study, the findings demonstrated a substantial reduction of cell damage by using an ophthalmic lens design of S-BF which was more superior to a UVF lens in preventing photodamage of RPE.

In recent years, LEDs have been increasingly employed for domestic lighting and in other commonly used electronic devices. LED lights are known to emit higher levels of blue light which may pose a threat to well-being, including retinal damage [19, 48, 49]. Increased exposure to blue light, particularly in the late evening or at night could potentially affect the circadian rhythm, which in turn disrupts melatonin secretion, the sleep-wake cycle, and mental alertness [50–53]. The application of a blue filter lens could reduce photodamage of retinal cells. However, this does not come without any disadvantages. The use of a full blue-blockage lens may alter colour perception, decrease scotopic vision, and also disrupt the circadian clock regulator by blocking the spectral wavelength of ipRGC input (480 nm) to the suprachiasmatic nucleus. Therefore, the transmittance of blue light should be carefully chosen such that the benefits to retinal health should not outweigh the overall well-being of an individual. In this way, the S-BF lens with 70% transmittance of light may reduce the effects of blue light induced photodamage without significantly altering the circadian rhythm. Nevertheless, the effects of S-BF lens on the circadian clock still need to be investigated.

In this study, the methodology had some shortcomings. In our experiment, the RPE cells were illuminated from below the culture plates, which means the light direction was from the basolateral to the apical side. This is not the natural direction when light enters our eyes. This may not interfere much with the interpretation of our results, because increased ROS production and cell death upon LED irradiation were observed no matter the light source was placed from above or below the culture plates [10, 25]. However, it would be more ideal to take this into account when designing future experiments. Besides, clear-bottom imaging plates with a solid black upper structure could have been used to reduce variability of light intensity across different wells. It would also be more ideal if the treated cells being irradiated to different wavelengths of light and the control cells in the dark were cultured in the same well plate. In addition, it has been reported that RPE cells may recover from light-induced stress [54]. Therefore, light-dark cycles could have been incorporated instead of continuous light exposure. Furthermore, it has been reported that the presence of photosensitisers such as riboflavin can influence light-dependent ROS generation [55, 56]. It would have been better if the changes in the culture medium caused by light exposure were examined. Finally, in real life, light-induced retinal damage is usually a chronic cumulative injury. However, the model in our current study was an acute phototoxicity injury model at a domestic lighting level. This may explain why some of our results were significant but rather subtle, such as differences in trypan blue assay and ROS production between S-BF and UVF lenses. As shown in our results, the primary RPE cells lost their phenotype upon repeated subculturing. This has limited our experimental setup for investigating chronic injuries. To study the chronic effect, a primary RPE cell line that can maintain their phenotype for a long time should be used or established. In addition, an animal model should be used to investigate the protective effect of different ophthalmic lenses under chronic LED light irradiation.

## Conclusion

To conclude, it was demonstrated that blue LED light induced significant cytotoxicity on RPE cells and that the use of an S-BF lens resulted in protective effects on cell survival upon photo-irradiation, which were mediated by a reduction of ROS and increased levels of antioxidant enzymes.

## Supporting information

S1 Raw images File containing all data underlying findings for the study. (PDF)
