# Supplementary material for: Selective blue-filtering spectacle lens protected primary porcine RPE cells against light emitting diode-induced cell damage
Source: PLoS One. 2022 May 24;17(5):e0268796. doi: 10.1371/journal.pone.0268796 (PMC9129023; doi:10.1371/journal.pone.0268796)

# Supporting Information

## Original images for western blots

The blots were incubated with anti-mouse or anti-rabbit IgG conjugated with horseradish peroxidase (Zymed Laboratories, San Francisco, CA, USA) and visualised by Pierce SuperSignal West Pico Chemiluminescent substrate (Thermo Scientific). The images were captured and analysed with an Azure Imaging System c600 (Azure Biosystems, CA, USA).

## RPE65 at 65kDa

RPE65 western blots from Fig 4 in the main text.

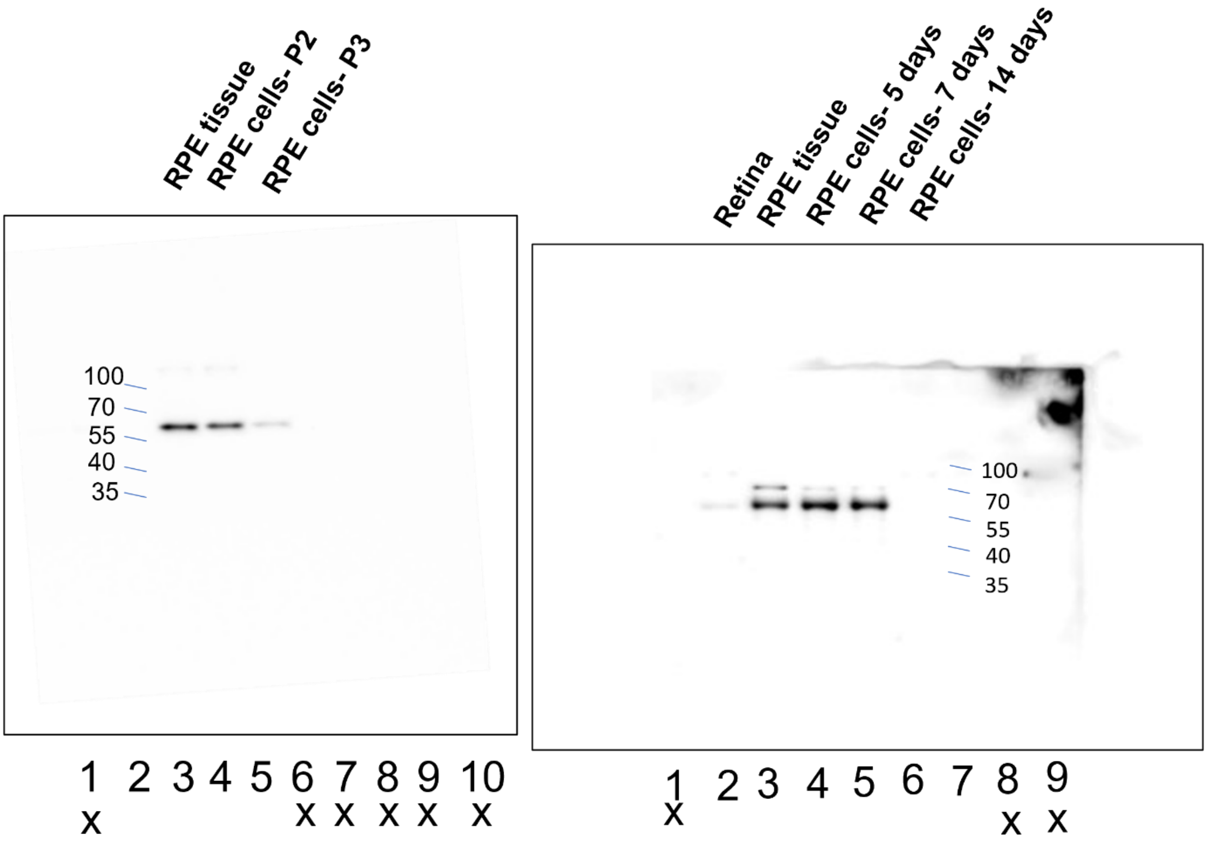

**β-Actin at 42kDa**

β-Actin western blots from Fig 4 in the main text.

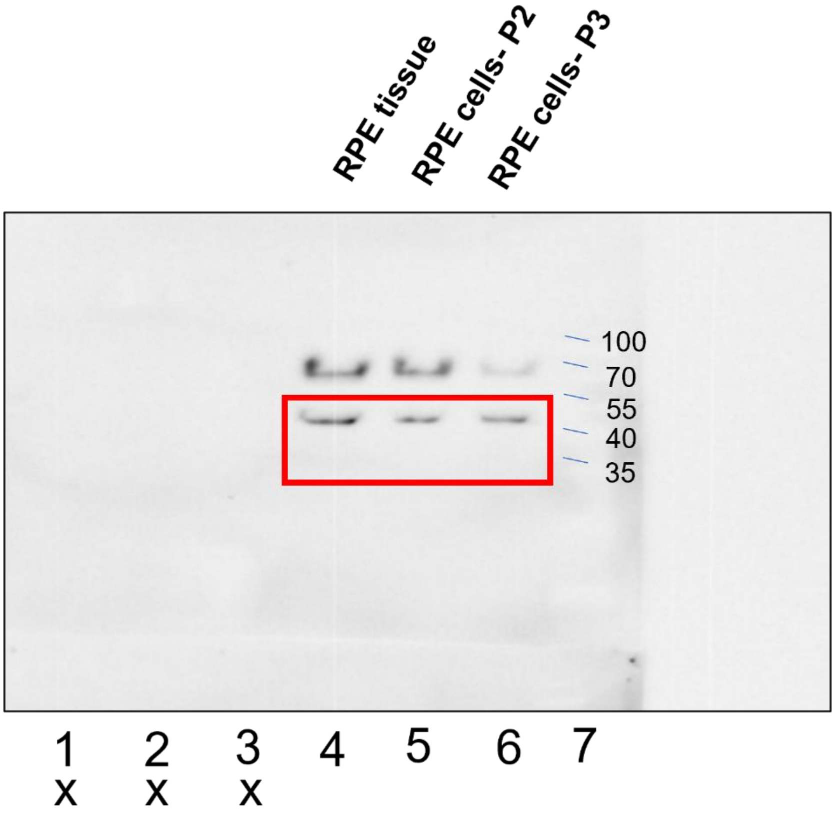


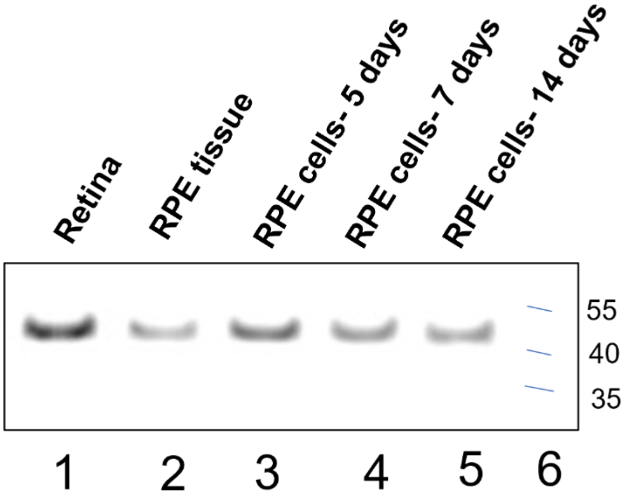

**Catalase at 59kDa**

Catalase western blots from Fig 9 in the main text.

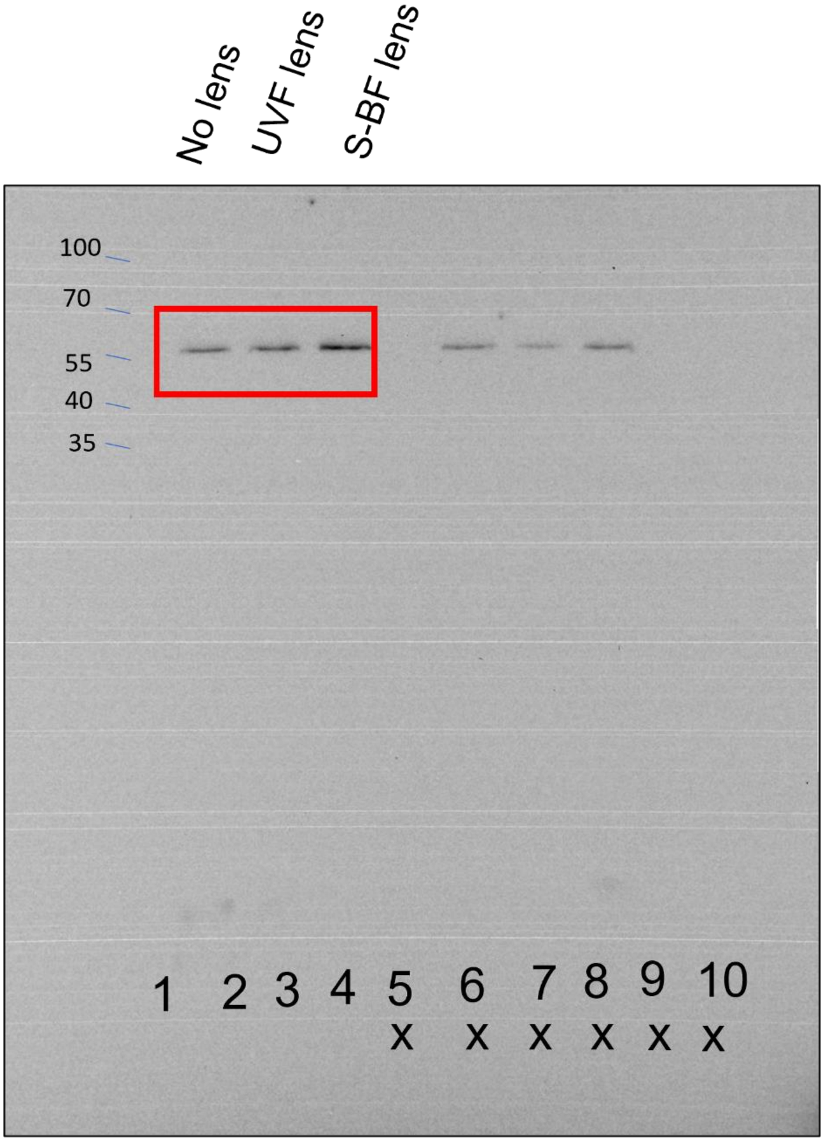

**GAPDH at 37kDa and Prdx3 at 28kDa**

GAPDH and Prdx3 western blots from Fig 9 in the main text.

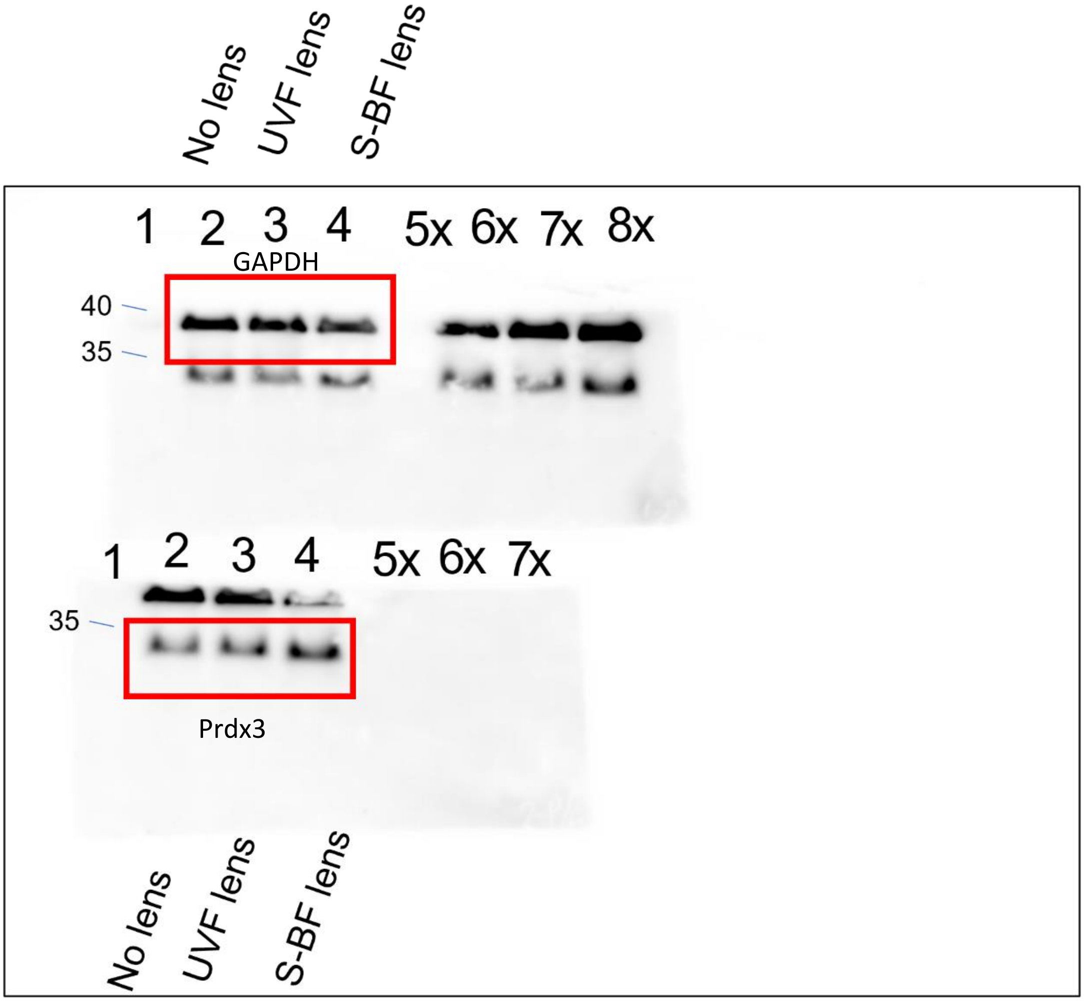

Supplement: S1 Raw images — (PDF) [file pone.0268796.s001.pdf]
